# Responses to gestational weight management guidance: a thematic analysis of comments made by women in online parenting forums

**DOI:** 10.1186/1471-2393-14-216

**Published:** 2014-06-30

**Authors:** Madelynne A Arden, Alexandra MS Duxbury, Hora Soltani

**Affiliations:** 1 Department of Psychology, Sociology & Politics, Sheffield Hallam University, Collegiate Crescent, Sheffield, UK; 2 Centre for Health and Social Care Research, Sheffield Hallam University, Collegiate Crescent, Sheffield, UK

**Keywords:** Internet-mediated research, Gestational weight gain, Parenting forums, NICE, Women, Views, Risk perception

## Abstract

**Background:**

The National Institute for Health and Clinical Excellence (NICE) published guidance on weight management in pregnancy in July 2010 (NICE public health guidance 27: 2010), and this received considerable press coverage across a range of media. This offered an opportunity to examine how gestational weight management guidance was received by UK women.

**Methods:**

A thematic analysis was conducted of 400 posts made in UK-based parenting internet forums in the week following the publication of the NICE guidance. This allowed us to examine the naturally occurring comments from 202 women who posted about the guidance on public forums.

**Results:**

Three main themes were identified and explored: i) Perceived control/responsibility ii) Risk perception iii) Confused messages.

**Conclusions:**

Women differed in their perceptions of the level of control that they had over being overweight with some feeling responsible and motivated to maintain a healthy lifestyle. Others felt there were multiple factors influencing their weight issues beyond their control. There were reports of feeling guilty about the impact of weight on the growing baby and experiencing significant obesity stigma from the public and health professionals. Information about the risks of overweight and obesity in pregnancy were difficult messages for women to hear, and for health professionals to deliver. Women reported being confused by the messages that they received. Health messages need to be delivered sensitively to women, and health professionals need support and training to do this. Risk information should always be accompanied with clear advice and support to help women to manage their weight in pregnancy.

## Background

In July 2010, NICE^a^ published guidance on weight management before, during and after pregnancy [1]. The publication of this document was widely publicised in the mass media, via television, radio, printed and online newspapers [2,3] The guidance emphasises that weight adjustment should be made mainly before pregnancy but also suggests that advice on healthy lifestyle and weigh management should be given during pregnancy. For health professionals, it outlines the importance of following approaches:

• Providing evidence based behaviour change advice including understanding consequences of health related behaviour, considering women’s social context and supporting women in planning health related behaviour changes with explicit coping strategies.

• Providing information on the health risks of obesity for mothers and their babies particularly for obese women.

• Advising on the healthy eating and physical activity including at least 30 minutes per day of moderate intensity activity.

Brown and Avery (2012) examined to what extent these guidelines have been implemented and suggested that a majority of women wanted information and advice on weight gain in pregnancy with a quarter feeling that there was a lack of advice and support [4]. Olander et al. [5] conducted focus groups with pregnant and postnatal women and health professionals and reported that women were generally unconcerned about their weight in pregnancy with an assumption that they would lose weight postnatally. Women also reported a reliance on the internet as a source of information when they did not receive it from health professionals. The health professionals in this study felt that they did not have sufficient information and skills to advise pregnant women due to the lack of national guidelines about gestational weight gain. A further, small scale study interview study of obese pregnancy and post-natal women who had not utilised a weight management service found that some women were offended by being referred to the service and perceived that they did not need help [6]. Thus there are mixed findings about women’s and health professionals’ responses to the health guidance and the concept of weight management in pregnancy.

While these kinds of studies offer some insights into he way in which guidance about weight management in pregnancy has been received and interpreted they do have significant limitations in that the data is being collected in focus groups and interviews conducted by researchers who are likely to be perceived as having certain views and expectations of participants. For example, Eisinga et al. [7] found that the Body Mass Index (BMI) of the interviewer differentially affected participants’ reporting of restrained eating behaviours in relation to their BMI. Public online discussion forums provide an opportunity to collect research data where ‘participants’ do not have any interaction with a researcher. Online forums work by one member posting a comment or question on a forum and then other members commenting on the original post or discussing other comments within a ‘conversation’ known as a thread.

Online discussion forums have become a popular means of communicating and receiving support in a wide range of domains. As noted by Coulson [8], the popularity of these forums may be in part due to them being accessible at all times when it is convenient to the user, and also due to the anonymity offered which may provide participants with opportunities to “discuss sensitive or ‘taboo’ issues with less risk…” (p.580). There are a large number of UK-based discussion forums including a considerable number aimed at parents, mothers and women of child-bearing age. The posts made within these forums offer the opportunity to assess ‘naturally’ occurring commentaries un-influenced by the research situation and the perceived need to be ‘politically correct’.

Forums are used by parents for emotional support [9] or advice ranging from physical/social aspects of life such as which car seat to buy to health related aspects such as whether they should feed peanuts to a toddler or how much weight they should gain. McDaniel et al. [9] reported that new mums spent on average 3 hours a day on a computer, mostly on social networking sites, their own or other mother’s blogs and forums and that the social support received from blogging improved feelings of connectedness and maternal wellbeing. Thus forums are commonly used by pregnant and postnatal women and are therefore potential sources of naturally occurring ‘conversational’ data.

The use of posts within public discussion forums as a source of research data has increased in recent years. Studies have included for example: living with irritable bowel syndrome [8]; HIV/AIDS support groups [10]; veganism [11,12] and the Measles Mumps and Rubella (MMR) vaccine [13]. To our knowledge, however, no other study has looked at formal or informal weight management advice or discussions on parenting forums in the UK.

Public reactions to health risk information have been varied and often associated with unpredictable consequences. For example, while the 1988 salmonella scandal resulted in a dramatic reduction in the purchasing of eggs [14], the longer but less high profile campaign around cholesterol and heart health, only resulted in a minimal reduction of egg sales. Frankel et al. [14] suggested that these effects may be the result of ‘lay epidemiology’, where individuals interpret health risks through the personal experiences of their friends and family and from interpreting information from a range of sources including the TV (and more recently the internet). Wahlberg and Sjoberg [15] concluded from their review of risk perception and the media that while the media have influence on general risk perception, judgements of personal risk are resistant to change and much more influenced by personal accounts and direct experiences. Thus it would be useful to ascertain how information about the risks of obesity in pregnancy and guidance for weight management in pregnancy were received and interpreted by women.

In summary, many mothers use the internet in their daily lives and so parenting forums are a tool for capturing their views and opinions about guidance on the management of weight in pregnancy, and the media’s portrayal of it. This study therefore aims to explore women’s perspectives about the weight gain guidance using spontaneous and naturally occurring comments made in posts on public parenting forums.

## Methods

### Ethics

This study was granted ethics approval from Sheffield Hallam University and adhered to the guidelines developed by the British Psychological Society [16]. Informed consent was not sought, due to the fact that the data was collected from open access websites so already in the public domain. Care has been taken to ensure that the members posting remain anonymous and comments cannot be traced back to the specific forums or individual members. We have decided not to disclose the names of the forums.

### Data collection

Online parenting forums were identified by typing the term “UK parenting forums” into the Google search engine on the 28th of July 2010, the date the NICE guidance was published. Twenty one UK based parenting forums were identified and systematically searched for any content relating to the NICE guidance, or the British media’s portrayal of it between 28th July 2010 and 4th August 2010.

Three forums contained content on the guidance, in eight threads. These threads became the data sources for this study, with 202 forum members contributing to 400 posts. Posts ranged from a couple of sentences, to a poem or a long detailed account of their own positive or negative experiences in pregnancy. For the purpose of this paper, we were specifically interested in the comments relating to the NICE guidance and the media’s portrayal of the guidance, rather than the women’s personal pregnancy experiences and our selection of posts was therefore limited to this.

All posts in relevant threads were selected and the data cut and pasted into a data document. All identifying characteristics were removed from the posts and usernames replaced with pseudonyms such as F3B10, which represents forum 3, thread B, poster 10. This is in line with BPS guidelines for conducting internet-mediated research to protect the poster’s identities [16,17].

Although the focus was mainly on women’s responses, this included posts from a journalist, a moderator and a few health care professionals who commented on their practice and care pathways. Responses from all types of posters were included in the data analysis, however all the quotations presented in the Results section were from women, who made the vast majority of responses.

The eight threads varied in length from eight posts to over 250 posts. In some threads each member posted once, however in others members posted on average two or three comments, although some members (usually the original poster) were particularly active.

Some socio demographic data was collected but was limited to what the members wanted to share, (e.g. England or Rotherham, mum of 2). All members were women, except for a male television journalist seeking contributors for their news bulletin that evening. The members ranged from those trying to conceive, those experiencing their first or later pregnancies, to mothers sharing their experiences of pregnancy from 20 years ago. Most women appeared to be based within the UK, with a few from America, Canada, Australia and Japan and some which did not indicate their geographic location.

### Data analysis

The data was analysed using thematic analysis based on the methodology outlined by Braun and Clarke [18]. The data was read and re-read to ensure thorough comprehension by all three authors. Next, extracts of data were coded to initial themes generated from the data. These initial themes and allocated data extracts were discussed and agreed among the authors. The initial themes were then compared and grouped into three non-overlapping broader themes with sub themes.

The themes have been illustrated with paraphrased extracts from the posts. Direct quotations have not been used as these may allow the data to be matched with the original source and thereby compromise anonymity. The themes have been developed from quotations across the sources of data (forum threads), illustrating that similar comments were made in each forum thread, from a range of parenting forums. We have taken the data extracts at surface meaning with description and interpretation.

## Results

### Three major themes

**Figure 1:**
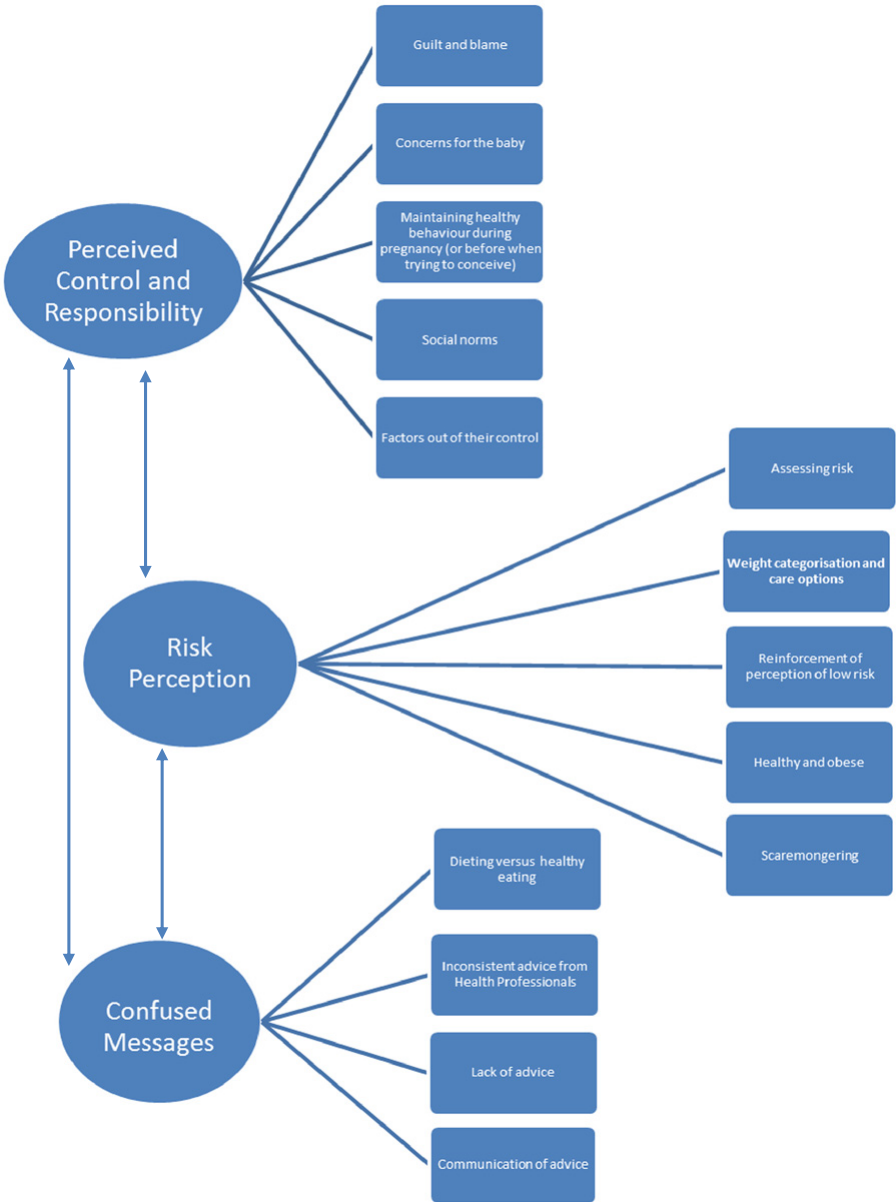
**Diagram of our three main themes and sub themes.** This diagram shows the three main themes (Perceived control and responsibility, risk perception and confused messages) with their sub themes. The arrows indicate how the three main themes are linked together.

Perceived Control and Responsibility, Risk Perception and Confused Messages were identified. These themes and sub themes are summarised in Figure 1 and will be discussed in turn.

### Perceived control and responsibility

The posters discussed a range of sources of perceived control and responsibility for being overweight or obese, and these were related to perceptions of blame and guilt.

### Guilt and blame

Within this sub-theme posters wrote about feeling selfish and responsible for the increased risks of being overweight or obese during pregnancy and that this was potentially impacting on their child. For some this was associated with feelings of guilt.

I suppose I am just selfish for wanting a baby even though I am fat.... (F3A49)

Others described feeling judged by others, including the medical profession.

I feel ugly and judged more than enough as it is,……without feeling that medical professionals are looking down their nose at me and seeing me as some selfish fat person who chooses KFC over a baby. (F3A45)

No woman should be made to feel guilty about her weight when pregnant, she should be guided and supported. It’s a tough enough time without going through it with a side order of guilt. (F3A42)

When I’m sick everyday and so only eat toast, I don’t want to be lectured about my weight and made to feel even more guilty. (F3A21)

Other posters expressed a view that the problem was the responsibility of the overweight person and that they should acknowledge the additional risks. They also felt in some cases that health professionals should be more explicit about informing overweight and obese individuals about the risks.

People seem to regard being overweight as a legitimate lifestyle choice - “It’s my body, I don’t see why I should have to lose weight” - but if you are pregnant, it may have long-term consequences on your children. I wish people would just face up to it and one way of doing that is if someone presents at a GP’s surgery saying “I can’t get pregnant”, doctors should have the courage to tell them their weight could be a factor. I‘ve spoken to some on this issue and the consensus seems to be that they don’t like to as it’s a sensitive issue. (F3B10)

Some posters related this responsibility to the limited resources of the NHS and the fairness of using those resources when the problem is under their control.

The NHS needs to spend its resources wisely and so it’s fair to expect people to make lifestyle modifications to improve their chances of a straight forward pregnancy. (F3A26)

### Concerns for the baby

Some women wrote of feeling bad that they could be harming their unborn child’s health and wellbeing, and so felt extra pressure to do everything perfectly during pregnancy.

I was diagnosed with Gestational Diabetes yesterday and cannot bare the guilt that I am feeling that my choosing to get pregnant even though I knew I was not in the “normal” BMI category may have health implications for my baby. (F3A49)

I truly wish I could be slim and toned and perfect BMI, but it won’t ever happen and I’m sick of the pressure. (F3C01)

### Maintaining healthy behaviour during pregnancy (or before when trying to conceive)

Some women were aware of the risks and complications of maternal obesity, and made efforts to control their weight before and during pregnancy.

I am now exercising and eating smaller portions to get my weight down to what it was when I last conceived. I don’t particularly enjoy having to do it but it makes sense, makes me feel better, and pregnancy is such a strain on the body that surely it’s good to be as fit as you can before you start trying to conceive. It must be such hard work for 9 months to be obese and carry a baby on top of that. (F3A48)

I gained 1 stone. I was slim before pregnancy and perhaps a bit too controlling. I have relaxed my eating but am keeping up my 5 mile swim, it makes me feel better. (F2C19)

I plan every week our food and have done all pregnancy to minimise weight gain. It’s so easy to reach for the fatty food and blame the baby. (F3A69)

### Social norms

Some women commented on societal expectations that all pregnant women should follow a healthy lifestyle by eating a balanced diet, abstaining from alcohol and cigarettes, exercising regularly and maintaining a healthy weight.

Women’s bodies are considered public property; something to be legislated about or publically shamed into different shapes. (F3A01)

Some reported that others felt able to make public judgements about dietary choices using the baby as a reason to justify this expression.

I have gone into work and had a comment made about everything I ate today (some celery, 2 apples and some ham salad sarnies on wholemeal, for the record - no cake, crisps or biscuits) - “you mustn’t eat for two you know, they said so on the telly”. (F3A66)

I had strangers touch my tummy without asking, all sorts of personal questions and even someone condemn me out loud in a supermarket for buying a prawn sandwich. (F3A74)

This may reflect a societal change in the acceptability of obesity in pregnancy. While smoking in pregnancy is considered unacceptable [19], views about overweight and obesity in pregnancy are less clear.

### Factors out of their control

Some women spoke about other issues in their lives which impacted on their ability to eat well and exercise in pregnancy. These included tiredness, morning sickness, caring responsibilities and work pressures.

……it’s not like women who have struggled with their weight all their lives don’t realise that it’s a bad idea to be massively overweight when pregnant - but like everything else, it’s not necessarily within your control. (F3A01)

I was going to have such a healthy pregnancy, eat only good food and be worthy, but then the sickness started, so I eat what I can keep down. (F3A25)

Lost a little weight so far, despite not being able to do my normal running, because it makes me sick. (F3A25)

Some women suggested that their weight was not in their control due to medical conditions such as auto-immune diseases or thyroid problems.

Smoking is a choice - weight sometimes isn’t. One of the treatments for my medical condition for instance is steroids for the rest of my life. (F3B04)

In summary this theme included a range of views about the relative responsibilities of obese or overweight women, before and during pregnancy. Some were critical of health professional’s failure to raise these issues. Others felt that in some instances the situation was not under the control of the overweight or obese person and therefore they should not be blamed. Underlying these comments were issues around societies’ reaction to obesity and stigma.

### Risk perception

The second theme related to women's perception and interpretation of the risks of overweight and obesity in pregnancy.

### Assessing risk

Some posters acknowledged and understood that they were at some risk and related the additional risks of being obese in pregnancy to other possible risks.

The doctors just said ‘yes, you have an increase for complications of about 50%, but all that means is that if 1 in a 100 women have a complication then 2 in a 100 at your size will have the same complication’. Put everything in perspective for me. (F3A38)

Some were questioning the route of the problem of obesity in pregnancy, in terms of complexity of contributing factors and their impact on pregnancy outcomes.

Is it because being overweight/obese causes medical problems in pregnancy? Or is it also because overweight or obese women are more likely to be poor, undereducated or from marginalised groups which always have riskier pregnancies? Both. (F3A61)

### Weight categorisation and care options

Some women felt their circumstances were not fully considered to make informed choices about their care options, due to their BMI category. They felt they were labelled in certain BMI categories which negatively impacted on their pregnancy experience.

My BMI was 34 when I got pregnant, so I am not allowed to go in the birthing centre (even though I exercise regularly, run 10 K races, eat healthy)… So far I have only put on a stone and will diet after birth to get my BMI under 30 just to avoid being put in this bracket in future as it is so irritating to have my choices removed. (F3A29)

They were specifically frustrated by the lack of flexibility to apply the guidelines to different settings, or to be able to holistically assess each woman individually.

I was annoyed at the ruling that midwife led units were out of bounds as NICE classes all MLUs together. I understand why I couldn’t go to one half an hour away from the obstetrician, but why wasn’t I allowed to go to one that’s one floor below labour ward?...... But if I had less confidence or a less strong birth partner, the obese protocol would make the birth harder and with possibly unnecessary interventions because health care professionals get caught up with protocol instead of looking at individuals. (F3A15)

Others questioned the categorisation of obesity by health professionals.

I am currently 17 stone. I am 5 foot 7. I am obese by their standards, my baby is healthy, I am healthy, and I won’t be dictated to! I listen to what my body needs. (F1A08)

### Reinforcement of perception of low risk

Many women posted about other individuals with a high BMI or large gestational weight gain who had healthy babies. These examples served to reassure them that they should be fine too.

Don’t worry about it! I was 40 and obese when I fell pregnant. I was weighed at my booking appointment, and no comment was ever made about my weight during my pregnancy…….. Thankfully it didn’t cause me any issues during pregnancy. After the birth I hadn’t actually put on much at all and I lost what I had by breastfeeding and lots of walking. (F3A44)

I gained 4 stone in pregnancy… everyone’s different, not to worry, if you are hungry-eat. (F2C04)

Thanks to everyone who has posted about their weight not being a problem - it’s nice to have some reassurance! (F2B07)

### Healthy and obese

Some women reported having a very positive self-image and felt that they were currently healthy and obese as having no existing medical conditions prior to pregnancy, so should stay healthy in pregnancy.

I’m morbidly obese but eat a healthy diet. (F3A09)

They reported that they do not consider their own obesity a negative health issue, when they still can live a normal life.

….for me obesity is a problem when you can no longer walk to the shop or tie your own shoe laces, NOT when you can still run with your dog and walk the length of the seafront daily, I am morbidly obese, yet I have No health issues. (F1A04)

### Scaremongering

Some women indicated a lack of trust in NICE to provide unbiased evidence, and a belief that there must be a NHS cost saving agenda.

It’s not just a question of individual behaviour - we live in a divisive, unequal society. And neither NICE nor the government dare wrap their heads around that. Much easier to blame the women. (F3A61)

There were also examples of comments indicating distrust for the media who have highlighted the increased risks and caused panic and guilt around the topic.

I saw this on the news this morning and thought it was a scare tactic more than anything! I don’t know if what they are saying is strictly true, as there are plenty of normal weight women who miscarry and loads of obese women who have perfect babies!!! (F2B04)

In summary, women did not accept the risks of overweight and obesity in pregnancy on face value. They questioned the validity of motives of the source of the information, the categorisation of women with a high BMI as ‘unhealthy’ and the impact of on their choice of care options, and focused on anecdotal accounts of positive outcomes.

### Confused messages

Women reported being confused by the kinds of messages they received and the way in which these messages were received.

### Dieting versus healthy eating

Some women wrote about their interpretations of the healthy eating message being about calorie control.

You just eat the recommended varied healthy diet and stick roughly to the calorie recommendations - for many people this will be lower than what they were eating pre-pregnancy, myself included. (F3A51)

I was good foodwise until pregnancy and have tried hard but crave fast food. I have been a bit naughty, as was living on 1500cals a day, 2000 now is bliss. (F2B09)

Others wrote about the quality and types of food as being more important.

I don’t care about my weight really, just the quality of food I eat. (F2C18)

But that even this was not always considered appropriate in pregnancy.

I lost a stone since becoming pregnant just by cutting out junk, had no morning sickness just cut out junk. Some people are saying “that can’t be healthy in pregnancy” well I believe it is healthier than to be snacking on fizzy drink, chocolate and crisps all day. Sometimes you can’t win! (F2B01)

### Inconsistent advice from health professionals

Comments indicated a lack of consistency in advice given by different health professionals. Some health professionals appear to be supporting their belief that being overweight is not harmful, by not mentioning it or normalising it.

I’m a large lady but even my midwife said most people are nowadays so as long as we try to eat as healthily as possible then we will be fine. (F2A04)

I can assure you that nothing has ever been mentioned to me about my weight in 9 months of antenatal appointments with midwives and consultants. (F3A12)

My midwife never weighs me, so I guess she’s not too worried either. (F2C11)

Or by not wanting to offend the mothers,

I raised the topic once and was advised that they no longer commented on weight unless there were actual and immediate health problems caused by it, as it only made women feel bad about themselves and during pregnancy was not the time to deal with it. (F3A44)

Some women mention they have had their raised BMI treated differently by a range of specialists.

My midwife at the booking in appointment wasn’t fazed at all by my BMI of 32, just said “try to eat healthily”. Then at my scan and bloods at the hospital it was totally different it was all “we have to monitor your weight carefully and if you go over 40 then you have to be under consultant care”. (F3A94)

I was informed of the risk of preeclampsia and diabetes by the consultant but he mentioned nothing about stillbirth and that’s the bit that panics me! I was told that I would have to be monitored more closely by the consultant, but when I saw him he said everything was fine and didn’t want to see me again until I was 34 weeks! So I guess I can’t be THAT much of a risk! (F2B26)

### Lack of advice

Some posts suggested that there is a lack of information and advice regarding ideal gestational weight gain with some reporting using online gestational weight gain calculators to fill this gap.

I typed my weight and height into a pregnancy weight estimator tool online and it told me I should gain 3 stone. I have gained approximately that now and am due. I’m convinced that half will fall off with the baby, placenta, water, blood etc. and the rest I will lose breastfeeding. (F2C15)

They also mention wanting personalised advice, rather than being judged purely on their pre pregnancy BMI.

I know NICE’s guidelines are what they are and fair enough. But whatever happened to personalised healthcare. It seems pretty bad to me that professionals should see which boxes a patient fits into and then decide on the best treatment or advice on that basis…….. I am a person not a statistic and I deserve healthcare advice that’s right for me, not my demographic. (F3A55)

There was mixed advice as to what to eat in pregnancy with some mentioning booklets, others mentioning a commercial slimming group which has a healthy eating plan for pregnancy, and others just airing their personal views about a healthy diet. They wanted more advice on how to cope with morning sickness and suitable exercises during pregnancy, asking each other for suggestions and sharing what has worked for them.

There was little practical advice in there about how you balance cravings, sickness and exhaustion with a varied diet and then to say when you do gain weight don’t try to lose it too fast. (F2B11)

They wanted clarity in the guidance and from their healthcare teams as to why being obese increased their risks and what they could do to reduce them.

I want to know the comparative risks of dieting while pregnant vs being overweight and pregnant. (F3A51)

I think what this guidance fails to address is why overweight/obese woman are at higher risk. (F3A61)

…. simply saying “you and your baby are at risk because of your weight” really doesn’t help address the issue. I have no idea what the solution is, but just warning about the risks clearly isn’t the answer! (F3A75)

### Communication of advice

Many reported that the health care professionals think obesity is just an energy imbalance, without considering other medical, psychological or social factors involved. They also questioned whether midwives had the time and resources to tackle obesity,

Then they can go on about effective weight management all they want. The reality is most of us know it, we find it hard to do and in the seven minutes or so a midwife has, she’s not going to break a lifetime of habits that have been too hard for the woman trying to be slim to break. (F3A68)

I think the “back up” advice is simplistic. The majority of women are well aware what constitutes a healthy diet. I find the tone of the document simplistic and patronising given the complexity of women’s issues with weight. (F3A68)

and repeatedly mentioned how they wanted to be treated without prejudice for being a ‘larger lady’.

They are there to advise but their main job is to support a lady no matter what shape/size she is and without prejudice. (F2C14)

They have said I need the blood pressure cuff “for larger ladies”. (F3A30)

Mainly I am saying that it is a good idea to raise awareness but as previous posters have said, maybe the way it is delivered is not great. (F3B03)

The tone of the guidance was repeatedly mentioned as being ‘anti fat’ and too patronising, comparing it to the abstaining from alcohol advice, or a ‘nanny state’.

We are no longer credited with having the brains or common sense to assess for ourselves the risks we are taking, and so they err on the side of not so much caution but absolute suppression……. If it has happened with booze, nuts, cheese, pate, liver and mayonnaise, why shouldn’t it happen with BMI? (F3A27)

I don’t know where they get their stats from but I am sure they are biased. Anti fat people. (F2B02)

In summary, there was confusion about what the guidance meant in terms of dietary and exercise behaviour, and a lack of consistency in how advice and messages are delivered by health care professionals. When weight, obesity and risks were not raised by health professionals or not done so in a consistent way, this served to reinforce a belief that the message was less important and therefore lowered perceptions of risk. Women wanted personalised, practical advice delivered sensitively.

## Discussion

The identified themes indicated that the response to the guidance on the gestational weight management was varied and complex. While some women acknowledged the importance of the guidance, and the lifestyle changes it recommends, others questioned the validity of the guidance and the reality of increased risk. There were also some views which defended the right of women to be obese and questioned whether this was necessarily related to poorer health. The messages that women reported receiving from a range of sources were confused and led to further questions about the reality of risk. The women also reported that awareness of increased risk was insufficient by itself and needed to be combined with useful, personalised advice about how they could manage weight in pregnancy.

The responsibility and control for obesity and the potential effects these might have on the health of mothers and infants were extremely varied, and this variation related to the extent to which individuals were perceived as being able to change their situation or to be blamed for it. This reflects views about the responsibility and control of obesity more broadly. Ogden et al. [20] reported that while patients were more likely to blame obesity on internal uncontrollable factors such as gland or hormonal problems, slow metabolism and stress, general practitioners were more likely to blame the amount of food consumed by the individual. This mirrors more widespread beliefs that obese individuals are responsible for being overweight and that weight gain or loss is under their personal control [21].

The perceptions that obesity is the responsibility of the individual are related to beliefs that obese individuals are lazy, undisciplined, with low will-power and are thus central to obesity stigma [21,22]. This stigma is widespread and extends into health-care settings, with negative attitudes towards obese patients being expressed amongst a wide range of health professionals including doctors, nurses, dieticians and fitness professionals [23]. These negative experiences were identified frequently by the women in our study and were experienced both from members of the public and health professionals. Negative attitudes towards obesity have been suggested to result in less than adequate provision of weight management guidance and advice by health professionals [23] and negative experiences of treatment by overweight and obese individuals [23]. Some women in our sample felt they received inappropriate comments or lack of advice regarding their weight and gestational weight gain, which is in line with findings from previous investigators [24-26]. Thus there is a significant need to address both obesity stigma and skills and confidence in providing weight management advice to overweight pregnant women amongst health professionals.

The women’s perceptions of risk in response to the guidance were varied and, in line with Wahlberg and Sjoberg [15], their personal risk perceptions reflected an emphasis on personal experiences and anecdotal accounts above statistics. Consistent with Sui et al. [27], we found some women were motivated to lead a healthy lifestyle; however barriers such as lack of time, insufficient advice and confidence in their ability to make health behaviour changes were identified. Many of the women attempted to minimise the threat of their increased risk consistent with a wide range of research showing that people tend to respond to personally relevant health information in defensive ways [28]. They used a range of strategies to do this: they questioned the quality of the information [28,29]; and they showed unrealistic optimism by focusing on aspects of their lives and behaviours which were ‘healthy’ to counteract the supposed risks [30-32] and by showing egocentrism in which they focused on factors which reduced their risk but failed to acknowledge that others may have as many or more factors which reduce their risk [32]. This downplaying of risk in response to threat has been widely described in a range of domains and poses a particular challenge to health professionals who are trying to communicate risk in order to promote behaviour change [33].

One particular issue raised in relation to risk perception was whether individuals and health professionals appropriately classified overweight and obese. If obese women don’t classify themselves as obese, then they will not engage with the messages around risk. Johnson et al. [34] compared weight perceptions between 1999 and 2007 and found that as the national average self-reported BMI had increased, less overweight people (75% in 2007 vs 81% in 1999) correctly classified themselves as overweight. With significant numbers of overweight and obese people not recognising their weight as a cause for concern, this could lead them to dismiss weight related health messages as not relevant. Burke et al. [35] compared self-perception of weight status between 1988–1994 and 1999–2004, and found that more overweight people were classifying themselves as ‘about right’ rather than ‘overweight’. This generational shift in social norms towards an acceptable higher bodyweight may result in people being less engaged in public health weight loss campaigns. This normalisation of overweight is a challenge for health professionals both in terms of identifying those at risk and explaining the classification and risks of obesity.

In line with previous findings, women reported being confused about what the health messages about weight management in pregnancy meant, and had received conflicting information from different sources [25]. While the NICE guidance was intended to provide a source of information for health professionals to facilitate clear messages to women, recent research has suggested that the awareness of this guidance amongst health professionals is lacking in some areas (Talking Health in pregnancy research, unpublished manuscript). A consistent finding was that women felt that they needed personalised messages to help make health behaviour changes i.e. *how* would they do this. This is consistent with evidence suggesting that merely proving information about risks is unlikely to result in behaviour change [36]. The women identified a range of barriers to weight management in pregnancy which included sickness, tiredness and food cravings. Efforts to support women to manage weight in pregnancy are likely to require a range of behaviour change techniques and not rely on providing risk information alone. This is in line with research showing that women would like motivating, supportive, non-judgemental care; ideally continuity of care from the same midwife throughout pregnancy to build rapport; and social interaction with other obese mothers [37].

The women in our study also acknowledged how difficult this would be for health professionals to deliver within the limited time available to them. Some mentioned looking elsewhere on the internet, to source weight loss solutions [38] and practical advice on healthy eating and physical activity [39]. However, given that the quality of information on the internet can be poor [40] there is therefore a need to provide women with additional services to complement and support healthcare professionals. Qualitative work by Furness et al. [37] and Soltani et al. [41] explored this need for additional support and gathered service user’s views which were predominantly positive regarding a prototype system using text messages and goal setting diaries to provide this healthy lifestyle support during obese pregnancies.

Study strengths and limitations.

As expected, the method of data collection allowed for the collection of a range of spontaneous views produced in a situation where individuals felt able to comment without the demand characteristics of a research situation and with the protection of anonymity [8]. This allowed us to assess honest public perceptions of the guidance about weight management in pregnancy. The other advantage of this method is being resource effective and allowing access to a large amount of data in a relatively short period of time. However, we had no control as to who could participate in the forums, and have no exact measure of their exposure to the guidance, (whether they read the whole guidance or just one or more newspaper articles on it, or heard it on the news bulletin). Thus whilst some posters may have been responding directly to the NICE guidance, others were reacting to the media’s portrayal of the NICE guidance. However, all posters were writing about the concept of ‘weight management in pregnancy’.

Due to the fact that posters can remain anonymous on forums, we do not have detailed information about the demographic characteristics of the sample. It is likely that the sample was somewhat biased towards middle class women who are known to be the most frequent users of internet forums [42]. This study was conducted in the UK using UK based forums, however a small proportion of the women posting were based overseas (Japan, Canada, Australia, America), so may be referring to the care they received in these health care systems which are very different to the NHS. In most, but not all cases the posters mentioned their own body size or BMI, often to justify their argument, but this was not the case for all posters so we cannot be sure of their weight status. However, while the characteristics and generalisability of the sample are not clear, the ability of this method to tap into spontaneous views is a clear advantage.

## Conclusions

In response to the gestational weight management guidance, there was a wide variation in women’s perceptions of risk, control, responsibility and understanding messages. Some women reported feeling guilty and experiencing significant stigma while many expressed a lack of control over their lifestyle and weight issues. This was partly related to a lack of support and information as well as associated communication issues. Information about the risks of overweight and obesity in pregnancy were difficult messages for women to hear, and for health professionals to deliver. Women reported being confused by the messages that they received. Health messages need to be delivered sensitively to women, and health professionals need support and training to do this. Risk information should always be accompanied with advice and support to help women to manage their weight in pregnancy.

### Implications for practice

By providing natural, honest and large scale data, the results from this study have important implications for developing or updating guidance in gestational weight management to inform health policy and practice. First, messages about the risks of obesity and overweight in pregnancy and support to help them to manage weight in pregnancy need to be delivered consistently and clearly across the different health professions who work with this group. Importantly, these messages should not be ignored or omitted, as the women in this study believed that if health professionals did not raise it as an issue, then it was not something that was relevant or significant to them. Second, health professionals need training to challenge obesity stigma so that they can raise these issues with women in a sensitive, non-judgemental way. Third, health professionals should not expect that providing women with knowledge about the risks of overweight and obesity in pregnancy is sufficient to allow them to change their behaviour and be able to manage their weight in pregnancy. There should be an awareness that women may not recognise that the risks relate to them and support offered to explain the risks to women sensitively but in a clear way. Information about the risks should not be presented alone, but should be accompanied with support to enable the women to make changes to her behaviour. It is likely that this support will need to be tailored to the specific needs of the woman. If this cannot be provided within the limited time resources available to health professionals then other means of support (e.g. websites, apps, text messaging) should be considered.

## Endnote

^a^The National Institute for Health and Care Excellence (NICE) is the independent organisation responsible for developing national guidance, standards and information on providing high-quality health and social care, and preventing and treating ill health. NICE guidance helps health, public health and social care professionals deliver the best possible care based on the best available evidence http://www.nice.org.uk/media/89C/8E/NICE_Charter.pdf.

## Abbreviations

NICE: National Institute for Health and Clinical Excellence; NHS: National Health Service; BMI: Body mass index; MMR: Measles mumps and rubella; BPS: British Psychological Society; MLU: Midwifery Led Unit.

## Competing interests

The authors declare that they have no competing interests.

## Authors’ contributions

MA designed the study, acquired the data, lead the analysis and interpretation of data, and drafting of the manuscript. AD contributed to the analysis, interpretation of data and data handling, and drafting the manuscript. HS contributed to the analysis and interpretation of the data, and drafting and critically revising the manuscript. All authors read and approved the final manuscript.

## Authors’ information

MA (BSc, PhD, C.Psychol, AFBPsS) is a Principal Lecturer and Reader in Psychology in the Department of Psychology, Sociology & Politics at Sheffield Hallam University. She is a Health Psychologist and Chartered Psychologist with a particular interest in health behaviour change and women’s health. HS (PhD, MMedSci, BSc, PgDip, RM) is a Professor in maternal and infant health and the Lead for Service Delivery and Commissioning theme at the Centre for Health and Social Care Research in Sheffield Hallam University, UK. She has been contributing to practice, education and research in midwifery for about 20 years. She is a member of Editorial Boards for maternity related journals, Research Standing Committee (RSC) for the International Confederation of Midwives (ICM) and Yorkshire &Humber Research for Patient Benefit Funding Committee. AD (BSc,MMedSci) is a Research Assistant working in maternal and infant health at the Centre for Health and Social Care Research in Sheffield Hallam University, UK. She is a registered Public Health Nutritionist with interests in maternal obesity, weight stigma and nutrition during pregnancy.

## Pre-publication history

The pre-publication history for this paper can be accessed here:

http://www.biomedcentral.com/1471-2393/14/216/prepub
